# The effects of hyperbaric oxygen therapy on insulin resistance—an approach to physiology

**DOI:** 10.3389/fmed.2025.1679615

**Published:** 2025-11-14

**Authors:** Mafalda Sampaio-Alves, Diogo Alpuim Costa, Inês Gomes-Alves, João Sérgio Neves

**Affiliations:** 1 Faculdade de Medicina, Universidade do Porto, Porto, Portugal; 2 Unidade Local de Saúde do Tâmega e Sousa, Penafiel, Portugal; 3 Oncology Functional Unit, Hospital de Cascais Dr. José de Almeida, Alcabideche, Portugal; 4 Haematology and Oncology Department, CUF Oncologia, Lisbon, Portugal; 5 Centro de Medicina Hiperbárica, Hospital da Luz Lisboa, Lisbon, Portugal; 6 Centro Hiperbárico de Cascais, Cascais, Portugal; 7 NOVA Medical School (NMS), NOVA Medical School, Faculdade de Ciências Médicas, NMS|FCM, Universidade NOVA de Lisboa, Lisbon, Portugal; 8 Comprehensive Health Research Centre (CHRC), NOVA Medical School, NOVA Medical School, Faculdade de Ciências Médicas, NMS|FCM, Universidade NOVA de Lisboa, Lisboa, Portugal; 9 Cardiovascular R&D Centre - UnIC@RISE, Department of Surgery and Physiology, Faculty of Medicine of the University of Porto, Porto, Portugal; 10 Department of Endocrinology, Diabetes and Metabolism, Faculty of Medicine of the University of Porto, Centro Hospitalar Universitário de São João, Porto, Portugal

**Keywords:** diabetes, endocrinology, hyperbaric oxygen, hyperbaric oxygen treatment, insulin resistance, physiology, hyperbaric oxygenation

## Abstract

**Background:**

Diabetes mellitus (DM) is a severe, chronic and complex metabolic disease that leads to multiple dysfunctions, including micro and macrovascular complications, which are a major cause of morbidity and mortality. Type 2 DM (T2D) is highly preventable, and the stages that precede it are the ideal target for therapeutic intervention. Hyperbaric oxygen therapy (HBOT) is an established medical treatment for several clinical conditions. Because DM is one of the most prevalent comorbidities in patients under HBOT, it has allowed the observation and inference of some of its effects on DM, suggesting clinical benefit in different spectrums of the disease. Our main aim was to gather the existing evidence on the impact of HBOT on insulin resistance, as this is the best predictor for the development of T2D.

**Materials and methods:**

The scoping review was the methodology chosen to include all available data. Exclusion criteria consisted of articles that did not mention the effects of HBOT on insulin resistance, described only the use of normobaric oxygen, or had no available translation to English, Spanish, or Portuguese. In addition, all data discussing any effects on insulin, insulin resistance, or insulin sensitivity were included.

**Results:**

Two hundred and thirty studies were found, and 17 were eligible. The HBOT appears to improve fasting glycaemia and decrease insulin resistance in patients with DM, with effects appearing after 1 treatment session. Additionally, it reduces levels of proinflammatory cytokines that contribute to insulin resistance. The duration of this sensitisation effect remains unknown, as do the contributing molecular factors.

**Conclusion:**

HBOT seems to improve glycaemic levels and insulin sensitivity, thus presenting a potential treatment approach to treat insulin resistance and its consequences. However, translation into clinical practice remains contingent on robust, yet unavailable, randomized clinical trials.

## Introduction

Diabetes mellitus (DM) is the most common severe metabolic disease in humans, with hyperglycemia as its main characteristic. Globally, it is the 8ᵗʰ leading cause of death, directly causing approximately 1.5 million deaths per year (1). By itself, hyperglycemia is responsible for an additional 2.2 million deaths (1), as it increases cardiovascular risk and also negatively contributes to other comorbidities.

Hyperglycemia results from an autoimmune process that selectively destroys pancreatic *β*-cells in type 1 DM (T1D). On the other hand, type 2 DM (T2D) is highly preventable and is preceded by a prediabetes phase, with a therapeutic window where intervention can cause the regression of disease mechanisms.

Several risk factors for T2D are well described, including insulin resistance. This is the best predictor of T2D development (2), and pertains to the relative impairment of insulin action in target tissues (e.g., liver, muscle, and adipose tissue). Insulin resistance disrupts glucose absorption in peripheral tissues and its hepatic synthesis (3). This phenomenon is frequently encountered in individuals before they develop T2D and seems to be associated with an increased prevalence of arterial hypertension, obesity, and dyslipidaemia. Insulin resistance is also a diagnostic criteria for metabolic syndrome (4).

Since T2D has shown a frightening tendency toward an increase in its prevalence, it is becoming increasingly important to focus on prevention and study the mechanisms of reversal and delay of the disease.

Hyperbaric oxygen therapy (HBOT) has seen a massive evolution in medical practice since the 20th century (5). Its physiological effects result from the increase in dissolved oxygen content.

Although T2D is not a formal indication for HBOT (6), this therapy is a valuable asset in the treatment of one of its consequences: diabetic foot ulcers (type 2 indication, level B evidence) (6).

The increasing number of diverse clinical conditions (decompression sickness, central retinal artery occlusion, sudden hearing loss, diabetic and atherogenic ulcers, late radiation-induced lesions, etc.) in which it has been used allowed us to understand that HBOT can affect human physiology far beyond what was initially expected.

The present manuscript discusses the most recent evidence on the effects of hyperbaric oxygenation on insulin resistance.

## Materials and methods

The scoping review, which followed the PRISMA criteria (7), was the methodology of choice to include all available data on HBOT’s effects on insulin resistance.

Queries were run on PubMed, Google Scholar, Cochrane Library and LILACS, employing <<“hyperbaric oxygenation” [MeSH] AND “insulin” [MeSH]>>. Exclusion criteria consisted of 1. articles that did not mention the effects of HBOT on insulin; 2. articles that described only normobaric oxygen use or exposure to hyperbaric air, not hyperbaric oxygenation therapy; 3. articles with no available translation to English, Portuguese or Spanish. All papers discussing any effect of HBOT on insulin, insulin resistance, or insulin sensitivity were included.

The database search was completed by the 9th of December 2024.

## Results

**Figure 1:**
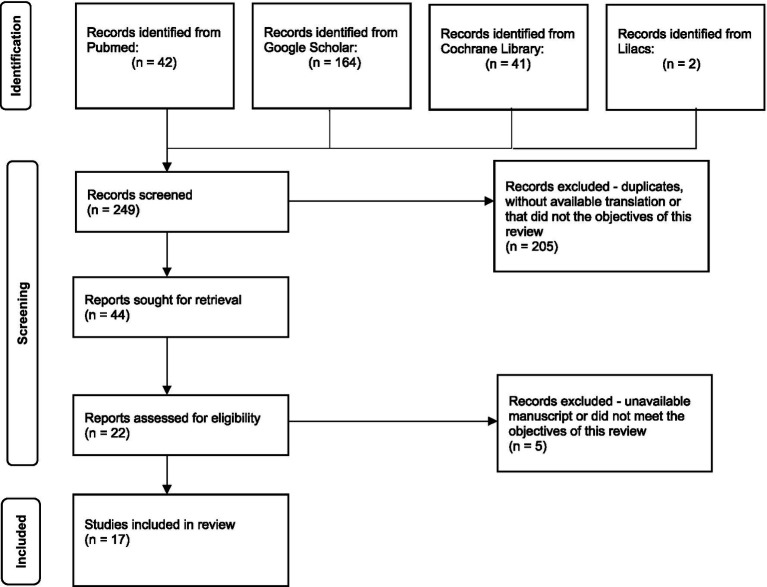
Adaptation of the PRISMA 2020 flow diagram for new systematic reviews (7).

In the primary identification phase (e.g., article searching), 249 results were obtained (Figure 1). In the screening phase, duplicates, articles with no available translations for the aforementioned languages, and those that did not fit the objectives of this review were excluded.

Ultimately, of the 44 articles independently assessed for eligibility by two authors (e.g., entirely read and analyzed), 17 were deemed eligible, excluding articles with no available manuscripts and those that did not meet the previously mentioned goals.

### Hyperbaric oxygen therapy

Hyperbaric oxygen therapy (HBOT) refers to the administration of pure oxygen (100%) for a brief period at a pressure higher than 1.4 ATA (absolute pressure). Its most frequent medical applications occur between 2.0 and 2.5 ATA, from 60 to 120 min (8). By using pressures higher than those found at sea level (1 ATA), the treatment sessions must occur in a hyperbaric chamber - a hermetically closed device where such pressures are obtained. The main goal of HBOT is to increase oxygen availability in different tissues.

Two fundamental commandments of physics are essential to understanding HBOT’s physiological effects: Dalton’s and Henry’s laws. Dalton’s law postulates that each gas exerts pressure in a gaseous mixture according to its proportion in the total volume. From this, it is inferred that oxygen constitutes approximately 21% of atmospheric gas and has a partial pressure of 160 mmHg. On the other hand, Henry’s law states that the concentration of a gas in a fluid will be given by pressure and its solubility coefficient. Therefore, the pressure exerted on a fluid by a dissolved gas differs from the pressure the same gas exerts on the gaseous phase. By applying the principle above, we know that, at T = 37 °C, carbon dioxide is 20 times more soluble than the oxygen molecule. 3,5 therefore, Dalton’s law allows us to know the partial pressure of each gas in a mixture, while Henry’s law allows us to assess the quantity of gas dissolved in a liquid.

Additionally, when addressing undersea and hyperbaric medicine, it is important to consider Boyle-Mariotte’s law, which states that a perfect gas at a constant temperature has an inversely proportional volume to its absolute pressure. This principle is extremely important as it can be used to steer recompression (e.g., for decompression sickness). Still, it also explains one of the main and most frequent adverse effects of HBOT (e.g., barotrauma).

In a physiological state, the human respiratory rate at rest ranges from 12 to 15 cycles per minute, with 6 to 8 liters of air inhaled per cycle. Given the percentages of gasses in the atmospheric mixture, we breathe approximately 250 mL of oxygen and 200 mL of carbon dioxide per minute. The passage of oxygen from the alveoli to the capillary occurs through facilitated diffusion and results from alveolar and ventilated oxygen pressure variation. As the variation in alveolar pressure of oxygen (104 mmHg) is constant, the only arm of this equation that can be modeled is the ventilatory rate. Thus, oxygen consumption can increase (up to 20-fold during strenuous physical activity) with no compromising diffusion, increasing capillary recruitment, vasodilation, and alveolar dilation.

After reaching arterial blood, 97% of our oxygen is transported by hemoglobin, and only 3% is dissolved in plasma, the latter referred to as “free oxygen.” Typically, 100 mL of blood carries 5 mL of oxygen to the peripheral tissues, where the partial pressure of oxygen is maintained at constant intervals. Even in strenuous physical activity, as this process causes a decrease in interstitial oxygen pressure, only 3 times as much oxygen can be transferred to tissues in the average person. Furthermore, when analyzing the sigmoidal shape of the hemoglobin-oxygen dissociation curve, it is evident that the affinity of hemoglobin for oxygen increases as the partial pressure of oxygen increases. In other words, the more oxygen molecules hemoglobin is bound to, the higher its affinity toward them.

Given the physical and physiological principles presented so far, it becomes evident that the physiological mechanisms from which HBOT’s effects arise do not target hemoglobin-bound oxygen but its fraction dissolved in plasma. Although the exact mechanism is yet to be clarified, it is known that under hyperbaric conditions, the partial pressure of oxygen in the plasma is 10 times higher (60 mL of oxygen per 1 L of plasma), satisfying the metabolic needs of the organism (9). This oxygen is more rapidly consumed in the different tissues, as it is not bound to hemoglobin. Jain (5), presents an interesting adaptation of the hemoglobin-oxygen dissociation curve, which shows that as pressure increases from normobaric (1 ATA) to different hyperbaric (1.5–3 ATA) environments, there are no changes in the quantity of oxygen bound to hemoglobin. Still, there is a linear increase in the partial pressure of oxygen.

### Adverse effects of hyperbaric oxygen therapy

Although the previously mentioned principles justify the benefits of HBOT, they also explain its most common adverse effects, which mainly include barotrauma and oxygen toxicity (5).

Boyle’s Law fundamentally defines the pathophysiology of barotrauma. It occurs when the pressure of an air-filled area cannot equalize with the ambient pressure. This pressure-related injury is frequent in phases with more significant pressure variations (e.g., descent and ascent phases) and occurs in entirely or partially non-collapsible cavities (10). Thus, the main affected sites include the middle ear, sinuses, lungs, intestines, teeth, eyes, and pathological spaces.

On the other hand, oxygen toxicity is believed to occur due to the body’s inability to eliminate excess oxygen free radicals. This complication impacts all tissues somewhat- the greater the inspired amount of oxygen, the earlier the manifestations appear. Seizures are a late sign of oxygen toxicity in the central nervous system (5, 7, 11). However, the frequency of these complications is significantly reduced when appropriate therapeutic protocols are applied. These should prioritize patient education (e.g., correctly performing Valsalva maneuvres during descent), judicious patient selection, and inclusion of proper air intervals (e.g., when the patient can stop inhaling hyperbaric oxygen inside the chamber) (5, 8, 10, 11).

### Physiology of insulin

Insulin is one of four hormones secreted by pancreatic *β*-cells. Discovered only in 1921, it regulates the metabolism of lipids, proteins, and carbohydrates in three primary target tissues: liver, muscle, and adipose tissue. Encoded by a single gene, the latter is transcribed and processed into pre-proinsulin, which is further cleaved into proinsulin. After removing the C peptide, an insulin molecule is formed. As this process occurs within the rough endoplasmic reticulum, this hormone is contained within a secretory granule, where it will associate itself with zinc. When glucose is stimulated, the *β*-cell releases a vesicle with insulin, proinsulin, and C peptide into the portal circulation.

Glucose is, therefore, the primary stimulus for insulin secretion. Its blood concentration (glycaemia) may vary in response to food intake or fasting, but healthy individuals do not have large fluctuations. Although to a lesser magnitude, β-cells also respond to galactose and mannose, which means that any of these three monosaccharides can induce insulin secretion. Nevertheless, glucose is the most potent secretagogue of insulin. Simply put, it enters the *β*-cell through GLUT2, where it undergoes glycolysis, increasing ATP concentration. This causes potassium channels to close and the cell membrane to depolarise, activating voltage-dependent calcium channels. With high calcium permeability, there is an influx of free calcium and an increase in its intracellular concentration, ultimately leading to insulin release.

It is relevant to consider the rich innervation of the islet of Langerhans by both divisions of the autonomic nervous system. This implies that *β*-adrenergic stimulation, where the vagus nerve releases acetylcholine, functions as an agonist of insulin secretion. On the other hand, in alpha-adrenergic stimulation, where the celiac nerves release noradrenaline, insulin secretion is inhibited. By increasing alpha-adrenergic activity, physical exercise constitutes an essential inhibitor of insulin secretion. Instead, food intake triggers multiple pathways that are ultimately stimulatory. In addition to this glucose-stimulating effect, there are enteric factors capable of increasing the β-cell’s response glucose per os, known as incretins: glucagon-like intestinal peptide 1 (GLP-1), cholecystokinin, and gastric inhibitory polypeptide (GIP).

After reaching the portal blood, during its first passage through the liver, 60% of the insulin is removed (3). Thus, insulinemia is not a reliable marker of the quantity of insulin secretion. In each target tissue, insulin will bind to its receptor (a tyrosine-kinase receptor) and propagate its signal through tyrosine phosphorylation. In the liver, it regulates key processes of energy metabolism. It affects glycogen metabolism, as increased insulinemia decreases the degradation and consumption of glycogen but promotes glycogenesis (formation of glycogen from plasma glucose). Here, insulin also promotes glycolysis and inhibits gluconeogenesis by promoting glucose metabolism. Regarding lipidic metabolism, this hormone stimulates lipogenesis, promotes lipid storage, and inhibits fatty acid oxidation. Moreover, insulin stimulates protein synthesis and inhibits protein degradation in the liver.

The remaining 40% that reach peripheral tissues will act on the muscle and adipose tissue. In these tissues, there is a different glucose transporter - GLUT4. In muscle, insulin stimulates GLUT4 (increasing the speed of glucose transport to the sarcoplasm) and enhances gluconeogenesis and glycolysis. Additionally, it also stimulates protein synthesis. In adipose tissue, insulin stimulates glucose absorption by recruiting more GLUT4 to the cell membrane and promoting glycolysis. In contrast to the liver and muscle, insulin promotes the use of glucose in synthesizing triglycerides and lipidic storage.

### Pathophysiology of insulin resistance

Insulin resistance occurs when this hormone fails to exert its effects at full capacity. The number of insulin receptors in each cell depends on new receptor synthesis, endocytosis and recycling, and endocytosis and degradation. A delicate balance between these three factors can maintain homeostasis, where despite the high number of receptors on the cell surface, there is a low recruitment rate.

However, cells chronically exposed to hyperinsulinemia are downregulated, experiencing a gradual decrease in insulin receptors and, consequently, a reduction in insulin sensitivity, even with its maximum effect (which occurs when, in a healthy individual, only 5% of the receptors are occupied) preserved (3). A practical example of this pathological phenomenon is observed in the adipocytes of patients with T2D, with fewer receptors per adipocyte area than healthy controls. In these cells, insulin resistance occurs, requiring a much higher concentration, even with a large fraction of occupied receptors (3). But, insulin resistance is a complex phenomenon that results from the sum of several malfunctions. Furthermore, it also includes impaired downstream receptor signaling, decreased insulin receptor activity, decreased PI3K activity, and possibly other steps that include recruitment of GLUT4 to the target cell plasma membrane (3, 12–16).

Insulin sensitisation is a much-desired effect of current therapies, whether behavioral (e.g., dietary changes and physical exercise) or pharmacological. As insulin and physical activity synergise by enhancing the recruitment of GLUT4 and increasing glucose oxidation, insulin sensitisation is a fundamental part of the therapeutic regimen of patients with DM.

### Effects of hyperbaric oxygen therapy on insulin resistance

While there is evidence in the literature that HBOT improves fasting glycaemia, declining it by 36% (16), and diminishing insulin resistance in diabetic patients, the mechanisms that account for the insulin-sensitizing effect of hyperbaric oxygenation still lack clarification. Vera-Cruz et al. interestingly postulate that hyperoxia’s acute blockage of carotid bodies might explain this effect, as these chemoreceptors are potent glucose and insulin sensors (17). Thus, as they can impact insulin sensitivity, their functional inhibition might account for the glucose tolerance observed in T2D patients exposed to HBOT.

Zhang et al. (18), inducing T2D in mice using streptozotocin and a high-fat diet, found that diabetic mice exposed to HBOT significantly decreased insulin resistance compared to non-exposed ones. Nevertheless, despite diabetic mice showing worse baseline pancreatic *β*-cell function, exposure to HBOT increased the cells’ area and volume, showing no differences, though no therapeutic effects were seen. Hyperbaric oxygenation seemed to decrease pancreatic β-cell apoptosis, which the authors hypothesized may contribute to its insulin-sensitisation effect.

In 2022, Sarabhai et al. again demonstrated that HBOT enhanced insulin sensitivity across all body tissues and reduced fasting blood glucose. The authors also described a discernible improvement in mitochondrial function, accompanied by stimulating reactive oxygen species (ROS) production and antioxidative defense in skeletal muscle and white adipose tissue (19).

In 2021, Kahraman et al. (20), proposed that the insulin sensitisation effect of HBOT was due to changes in the expression of resistin, PAI-I, and adiponectin. These adipokines are intrinsically associated with insulin sensitivity: high levels of resistin and PAI-I are associated with insulin resistance, while high levels of adiponectin are associated with insulin sensitivity. After submitting mice to hyperbaric oxygenation at 2,5 ATA for 20 consecutive days, they encountered a 7-fold increase in adiponectin mRNA compared to the other adipokines, raising the hypothesis that the effect of adiponectin is dominant over the others.

In another study, Wilkinson et al. (21), proved that HBOT’s insulin-sensitizing effect only occurs when using hyperbaric oxygen and not in hyperbaric air. Thus, the high partial oxygen pressures needed for this effect are only obtainable in an HBOT setting. Merely1 session of HBOT is enough to significantly increase peripheral insulin sensitivity, which persists for at least 30 min after exiting the hyperbaric chamber (21, 22). This effect is not exclusive to diabetic patients (22).

Moreover, when trying to evaluate the combination of stem cell therapy with hyperbaric oxygenation therapy, Xu et al. (21), discovered that HBOT also lowers the level of pro-inflammatory cytokines, such as TNF-alpha and IL-6. These are key factors for inflammation initiation and maintenance and are upregulated in T2D individuals, thus contributing to insulin resistance. Nevertheless, the impact of HBOT will depend on the severity of insulin resistance (23).

Hyperbaric oxygenation also seems to significantly reduce glycaemia and HbA1C, with increased C-peptide secretion. It decreases the dose of oral hypoglycaemic drugs and insulin, translating to improving metabolic control and *β*-cell function (24–29). When studying the effect of HBOT with autologous bone marrow stem cell transplantation, Wang et al. (28), concluded that this dose-decreasing effect was mainly seen in patients with shorter duration of disease and/or on lower doses of hypoglycaemic drugs. Additionally, pancreatic β-cell function only seems to improve transiently. Nevertheless, in a randomized controlled trial, when evaluating the combination of autologous bone marrow mononuclear cell infusion and HBOT, Wu et al. (30), observed that these results were not verified in the group exposed to HBOT alone (Table 1).

**Table 1:** Summary of the case series or clinical trials described in the literature regarding the effects of hyperbaric oxygen therapy on glucose metabolism.

Classification	Model	Reference	Population	Protocol	Results
Pre-clinical	Animal models	Goto et al., 2020, (12)	24 OLEFT + 22 LETO mice	Evaluation of the haemodynamic response during HBOT at 1.3 ATA, using muscle and oral glucose tolerance test at 48 h, in mice of different ages.	HBOT increased not only peripheral oxygen saturation, but also total and oxyhemoglobin in both groups. HBOT decreased deoxyhaemoglobin in both groups. Total and oxyhaemoglobin variations were higher in the OLEFT group. Variations in hemodynamic response under HBOT were significantly higher in the 8-week-old OLEFT group.
		Gu et al., 2010, (14)	20 male Goto-Kakizaki mice	Subjects were randomly divided into four groups: normobaric (NN), hyperbaric-to-normobaric (HN), normobaric-to-hyperbaric (NH) and hyperbaric (HH) groups.	HN and HH groups exhibited greater reductions in blood glucose levels compared to the NN and NH groups. HN, NH, and HH groups exhibited greater reductions in blood glucose levels than those in the NN group at 13-weeks. HN’s insulin levels were lower than those in the NN group. HH showed lower insulin levels compared to both the NN and NH groups.
		Matsumoto et al., 2007, (15)	10 male Winstar + 10 male Goto-Kakizaki mice	Mice were randomly sorted into a control group and an HBOT group. The latter group was submitted to HBOT at 1.25 ATA, 6-h-a day at 36% oxygen, for 4 weeks.	Goto-Kakizaki mice had lower plasma insulin levels in in the HBOT group.
		Yasuda et al., 2007, (16)	10 male Winstar + 10 male Goto-Kakizaki mice	Mice were randomly sorted into a control group and an HBOT group. The latter group was submitted to HBOT at 1.25 ATA, 6-h-a day at 36% oxygen, for 4 weeks.	Fasting plasma glucose levels of Wistar and Goto-Kakizaki mice were significantly lower in the HBOT groups. Fasting plasma immunoreactive insulin levels were significantly lower (*p* < 0.05) in the HBOT group in Goto-Kakizaki mice.
		Zhang et al., 2022, (18)	24 male C57BL/6 J mice	Mice were randomly sorted into four groups: High-fat-diet (HFD); HFD + HBOT; T2D and T2D + HBOT. The HFD and T2D groups served as control groups.	In T2D mice, HBOT lowered fasting blood glucose levels (not statistically significant). HBOT significantly reduced peak blood glucose levels at 15 min (*p* < 0.05). The HOMA-IR was significantly lower in the T2D + HBOT group when compared to the T2D group (*p* < 0.01).
		Kahraman et al., 2021, (20)	16 male Sprague–Dawley mice	Mice were divided into control and HBOT groups and submitted to similar feeding and housing conditions. The latter group was subjected to 20 days of HBOT at 1.5 ATA at 100% oxygen.	Fasting plasma glucose levels were lower in the experimental group (*p* = 0.016). HDL-C was higher in the experimental group (*p* = 0.012). Fasting insulin levels were lower in the experimental group (*p* = 0.013). HOMA-IR was lower in the experimental group (*p* = 0.012). QUICKI scores were higher in the experimental group (*p* = 0.012). Serum PAI-I levels were higher in the experimental group (*p* = 0.027). Resistin (*p* = 0.002), adiponectin (*p* = 0.002) and PAI-I (*p* = 0.001) levels in the retroperitoneal adipose tissue were higher in the experimental group.
		Xu et al., 2020, (23)	6 Male Sprague Dawley (SD) mice + 24 Male (SD) mice with T2D	T2D mice were randomly assigned to four groups: stem cell therapy (SC); hyperbaric oxygen treatment (HBOT); stem cell therapy and hyperbaric oxygen treatment (SC + HBOT); diabetes control group (DM); normal control group (NC).	Mice treated with SC + HBOT or SC alone demonstrated reductions in blood glucose levels, HOMA-IR, serum LDL-C and triglycerides, 24-h urinary protein, and serum TNF-*α* and IL-6 levels. Mice submitted to SC + HBOT or SC had an increase of the serum insulin secretion. Therapeutic effects were observed earlier in the SC + HBOT in comparison to SC.
		Juang et al., 2002, (25)	Male inbred C57BL/6 mice	Following the transplantation of an insufficient number of islets, mice were divided into five groups: (A) HBOT once daily for 28 days; (B) HBOT twice daily for 28 days; (C) HBOT once daily from day 5 to day 28; (D) HBOT twice daily from day 5 to day 28 and (E) a control group.	At post-operative day 28, blood glucose levels in groups B, C, and D were reduced in comparison to baseline measurements. Insulin content in groups B and D’s grafts was higher when compared to the control group.
Clinical	Human studies	Sarabhai et al., 2023, (19)	15 men, with T2D, HbA1c between 6–9%, BMI < 35 kg/m^2^	Patients were randomly divided into two groups: recipients of a single session of HBOT and a control group.	Fasting glucose levels decreased (*p* < 0.05) in the HBOT group. Whole-body, hepatic and white adipose tissue insulin sensitivity improved with HBOT (*p* < 0.05).
		Wilkinson et al., 2020, (21)	25 men with T2D, > = 40 years old, BMI > = 25 Kg/m^2^	Patients were randomly assigned to two groups: HBOT and hyperbaric air.	HBOT demonstrated an increase in glucose infusion rates, with a mean increase of 26% (*p* = 0.04) at steady-state 1 and 23% (*p* = 0.018) at steady-state 2. At steady-state 1, patients treated with HBOT exhibited significantly higher glucose infusion rates compared to those treated with HA (*p* = 0.036).
		Wilkinson et al., 2020, (22)	22 men, >18 years old, BMI > 25 Kg/m^2^, without T2D	Two studies were performed: 10 patients were submitted to hyperinsulinaemic-euglycaemic clamp to test insulin sensitivity during one HBOT session. 12 patients sustained frequently sampled intravenous glucose tolerance tests (FSIGT) to assess insulin sensitivity during and after 24-h of HBOT.	In the group where hyperinsulinaemic euglycaemic glucose clamp test was performed there was: Increased insulin sensitivity on day 1 of HBOT (*p* = 0.02). No statistically significant differences in the 30 min after one session. There were no statistically significant differences in the group who underwent FSIGT.
		Estrada, et al., 2008, (24)	25 patients with T2D, >18 years old	Patients underwent a combination therapy with intrapancreatic autologous stem cell infusion (ASC) + HBOT administered before and after the ASC procedure. Each patient received 5 consecutives daily HBOT sessions both prior to and following ASC infusion.	Mean BMI reduced during the follow-up (not statistically significant). Mean fasting glucose levels and mean HbA1c values significantly decreased in all patients during the follow-up (*p* < 0.0001). Mean C-peptide and mean C-Peptide/Glucose Ratio levels improved, peaking at 12 months following the combined therapy. In insulin-dependent patients using, the mean daily insulin requirement significantly decreased (*p* < 0.004).
		Chen, et al., 2007, (26)	31 patients with T2D + 29 healthy patients	Patients were submitted to HBOT at 2.5 ATA for 90 min per day during 3 consecutive days.	At baseline and after the 1st and 3rd exposures of HBOT. T2D patients had significant greater concentrations of HbA1C. T2D patients had significant lower concentrations of IGF-1. After the 1st and 3rd HBOT treatment, T2D patients had significantly greater insulin, leptin and NO serum concentration.
		Estrada, et al., 2019, (27)	23 T2D patients; BMI < 35 Kg/m^2^	Patients were randomly assigned to 2 groups: Recipients of combination of intrapancreatic ASC, HBOT, and standard medical treatment (SMT) OR control group. Each patient underwent 10 sessions of HBOT, before and after the intrapancreatic ASC.	HbA1c was significantly lower in the intervention group throughout the follow-up period, with the most pronounced difference observed at 180 days (*p* = 0.0025). Glucose levels were significantly lower in the intervention group during the entire follow-up, with the greatest difference noted at 90 days (*p* = 0.0000). C-peptide levels were significantly higher in the intervention group across all follow-up time points. At 90, 180, 270, and 365 days the intervention group required significantly less insulin.
		Wang et al., 2011, (28)	18 men and 13 women with T2D	All patients underwent a combination of autologous bone marrow stem cell transplantation (BMT) and HBOT during the peri-transplantation period. HBOT was administered five days before and after the stem cell infusion.	Mean HbA1c levels significantly decreased in all patients after BMT (*p* < 0.001) throughout all follow-up periods. C-peptide levels showed a significant increase at 90 days (*p* < 0.001) and remained consistent at other time points. All patients experienced a reduction in their hypoglycaemic therapy dosage, including insulin and/or oral medications.
		Xu et al., 2017, (29)	52 patients with T2D, >18 yo, diagnosed with intracerebral hemorrhage.	Patients were randomly divided into 2 groups: recipients of HBOT and the normobaric oxygen therapy (NBOT). The sessions were conducted daily for 1 month.	After HBOT, insulin sensitivity improved, and serum insulin, fasting glucose, and hemoglobin A1C were reduced (*p* < 0.05).
		Wu et al., 2014, (30)	80 patients with T2D, BMI < 35 kg/m^2^	Patients were randomly divided into 4 groups: recipients of bone marrow mononuclear cells (BM-MNC) infusion with HBOT (BM-MNC.HBOT group); recipients of only BM-MNC infusion (BM-MNC group); only HBOT (HBOT group) and control group. In the BM-MNC.HBOT group, 20 sessions of HBOT were included before and after BM-MNC infusion.	In both the BM-MNC.HBOT and BM-MNC groups, the area under the curve of C-peptide showed significant improvement, accompanied by a significant reduction in HbA1c levels. No notable changes were observed in the HBOT group. The insulin dose (IU/kg per day) significantly decreased in the BM-MNC.HBOT and BM-MNC groups, while no substantial improvement was observed in the HBOT group.

OLEFT, Otsuka Long-Evans Tokushima fatty mice; LETO, Long-Evans Tokushima Otsuka mice; HBOT, Hyperbaric oxygen therapy; HDL-C, high-density lipoprotein cholesterol; HOMA-IR, homeostatic model assessment of insulin resistance; QUICKI, quantitative insulin sensitivity check index; PAI-I, plasminogen activator inhibitor-I; FSIGT, frequently sampled intravenous glucose tolerance test; IGF-1, insulin-like growth factor 1.

## Discussion

Despite HBOT having been used therapeutically for more than a century, many of its effects are still unknown and poorly studied. T2D is the perfect paradigm of a disease that is sufficiently common for patients undergoing HBOT to have, but little is known regarding this therapy’s effects. This review shows that HBOT can improve fasting glycaemia, diminish insulin resistance and lower the levels of pro-inflammatory cytokines. With these effects appearing as early as the first treatment session, there is a need to explore this subject further.

Although there has been a gradual gathering of evidence that supports more significant investment in the study of HBOT as a complementary treatment modality for T2D, there continue to be several gaps that need more precise elucidation. In addition to clarifying the exact molecular mechanisms involved in sensitisation effects, evidence from randomized, double-blind, and placebo-controlled clinical trials should be privileged to guide clinical practice.

Further research should also try to understand and clarify inconsistent results between different studies (26). A shared aspect of the various available results is the inability to determine the duration of this sensitizing effect. Additionally, experiments have not yet ascertained which effects are transient and chronic, and with T2D being a chronic condition, efforts must be made to optimize these therapeutic effects for long periods. The ability to clarify this issue and determine specific timings of action and duration will considerably contribute to HBOT’s inclusion in T2D and metabolic syndrome therapy, as well as in the prevention of insulin resistance in patients with other major risk factors.

A specific aspect deserving special attention is the transversal lack of inclusion of women in human studies. This situation merits investment in future research, as it is essential to ascertain whether the observed benefits of HBOT are maintained in females, especially considering the endocrinological particularities that distinguish and might influence results between both sexes (22). Thus, sex-specific analyses are recommended. Additionally, any implications for clinical practice should ultimately be derived from robust randomized clinical trials, which are currently lacking in this area.

This review is subject to inherent limitations, most notably the substantial heterogeneity of the included studies, spanning preclinical and clinical designs, human and animal models, and varying experimental protocols. Additionally, no formal risk of bias assessment was performed. These methodological concessions were intentional, aiming to collate the entirety of available evidence in this field, thereby facilitating the identification of knowledge gaps and guiding future research directions.

Regarding the importance of including HBOT in the therapeutic regimen of patients with T2D, the current availability of other effective, less time-consuming and more low-cost treatment options is recognized. Nevertheless, none of these therapies has been shown to reverse insulin resistance – this remains a therapeutic target for which no effective intervention is currently available. Additionally, HBOT is associated with known adverse effects, which are outlined in this review. However, with current protocols and safety measures, such events have become infrequent.

Furthermore, because of its many therapeutic indications, HBOT has the potential ability to treat multiple diseases at once, decreasing the need/dosage for medications with more serious side effects. The ideal future of research in this field should investigate whether early intervention with HBOT in patients with insulin resistance has the power to reverse this condition in the long term and prevent its complications - something that the available pharmacological therapy cannot yet guarantee.

## Conclusion

The HBOT seems to improve glycaemia levels and insulin sensitivity, thus making it a weapon worth investigating in the fight against insulin resistance and its consequences (e.g., metabolic syndrome and T2D). Current literature presents enough evidence to support further and firmer investigation in this field, even with current optimized medical treatment. However, translation into clinical practice remains contingent on robust, yet unavailable, randomized clinical trials.

## References

[1] World Health Organization. Global report on diabetes. Geneva: World Health Organization (2016).

[2] Warram JH Martin BC Krolewski AS Soeldner JS Kahn CR. Slow glucose removal rate and hyperinsulinemia precede the development of type II diabetes in the offspring of diabetic parents. Ann Intern Med. (1990) 113:909–15. doi: 10.7326/0003-4819-113-12-909, PMID: 2240915

[3] Boron W Boulpaep E. Medical physiology. Philadelphia: Elsevier (2017).

[4] Huang PL. A comprehensive definition for metabolic syndrome. Dis Model Mech. (2009) 2:231–7. doi: 10.1242/dmm.001180, PMID: 19407331 PMC2675814

[5] Jain K. Textbook of hyperbaric medicine. New York: Springer (2017).

[6] Mathieu D Marroni A Kot J. Tenth European consensus conference on hyperbaric medicine: recommendations for accepted and non-accepted clinical indications and practice of hyperbaric oxygen treatment. Diving Hyperb Med. (2017) 47:24–32. doi: 10.28920/dhm47.1.24-32, PMID: 28357821 PMC6147240

[7] Page MJ McKenzie JE Bossuyt PM Boutron I Hoffmann TC Mulrow CD . The PRISMA 2020 statement: an updated guideline for reporting systematic reviews. BMJ. (2021) 372:n71. doi: 10.1136/bmj.n71 33782057 PMC8005924

[8] Alpuim Costa D Amaro CE Nunes A Cardoso JS Daniel PM Rosa I . Hyperbaric oxygen therapy as a complementary treatment for radiation proctitis: useless or useful? - a literature review. World J Gastroenterol. (2021) 27:4413–28. doi: 10.3748/wjg.v27.i27.4413, PMID: 34366613 PMC8316904

[9] Leach RM Rees PJ Wilmshurst P. Hyperbaric oxygen therapy. BMJ. (1998) 317:1140–3. doi: 10.1136/bmj.317.7166.1140, PMID: 9784458 PMC1114115

[10] Mathieu D. Handbook on hyperbaric medicine. Cham: Springer (2010).

[11] Costa DA Ganilha JS Barata PC Guerreiro FG. Seizure frequency in more than 180,000 treatment sessions with hyperbaric oxygen therapy - a single Centre 20-year analysis. Diving Hyperb Med. (2019) 49:167–74. doi: 10.28920/dhm49.3.167-174, PMID: 31523791 PMC6884101

[12] Goto N Fujita N Nino W Hisatsune K Ochi R Nishijo H . Hemodynamic response during hyperbaric treatment on skeletal muscle in a type 2 diabetes rat model. Biomed Res. (2020) 41:23–32. doi: 10.2220/biomedres.41.23, PMID: 32092737

[13] Ekanayake L Doolette DJ. Effects of hyperbaric oxygen treatment on blood sugar levels and insulin levels in diabetics. SPUMS J. (2001) 31:16–20.

[14] Gu N Nagatomo F Fujino H Takeda I Tsuda K Ishihara A. Hyperbaric oxygen exposure improves blood glucose level and muscle oxidative capacity in rats with type 2 diabetes. Diabetes Technol Ther. (2010) 12:125–33. doi: 10.1089/dia.2009.0104, PMID: 20105042

[15] Matsumoto A Nagatomo F Yasuda K Tsuda K Ishihara A. Hyperbaric exposure with high oxygen concentration improves altered fiber types in the plantaris muscle of diabetic Goto-Kakizaki rats. J Physiol Sci. (2007) 57:133–6. doi: 10.2170/physiolsci.SC000707, PMID: 17349108

[16] Yasuda K Adachi T Gu N Matsumoto A Matsunaga T Tsujimoto G . Effects of hyperbaric exposure with high oxygen concentration on glucose and insulin levels and skeletal muscle-fiber properties in diabetic rats. Muscle Nerve. (2007) 35:337–43. doi: 10.1002/mus.20692, PMID: 17094100

[17] Vera-Cruz P Guerreiro F Ribeiro MJ Guarino MP Conde SV. Hyperbaric oxygen therapy improves glucose homeostasis in type 2 diabetes patients: a likely involvement of the carotid bodies. Adv Exp Med Biol. (2015) 860:221–5. doi: 10.1007/978-3-319-18440-1_24, PMID: 26303484

[18] Zhang C Zhang D Wang H Lin Q Li M Yuan J . Hyperbaric oxygen treatment improves pancreatic β-cell function and hepatic gluconeogenesis in STZ-induced type-2 diabetes mellitus model mice. Mol Med Rep. (2022) 25:90. doi: 10.3892/mmr.2022.12606, PMID: 35039874 PMC8809048

[19] Sarabhai T Mastrototaro L Kahl S Bönhof GJ Jonuscheit M Bobrov P . Hyperbaric oxygen rapidly improves tissue-specific insulin sensitivity and mitochondrial capacity in humans with type 2 diabetes: a randomised placebo-controlled crossover trial. Diabetologia. (2023) 66:57–69. doi: 10.1007/s00125-022-05797-0, PMID: 36178534 PMC9729133

[20] Kahraman C Yaman H. Hyperbaric oxygen therapy affects insulin sensitivity/resistance by increasing adiponectin, resistin, and plasminogen activator inhibitor-I in rats. Turk J Med Sci. (2021) 51:1572–8. doi: 10.3906/sag-2011-76, PMID: 33705641 PMC8283499

[21] Wilkinson DC Chapman IM Heilbronn LK. Hyperbaric oxygen but not hyperbaric air increases insulin sensitivity in men with type 2 diabetes mellitus. Diving Hyperb Med. (2020) 50:386–90. doi: 10.28920/dhm50.4.386-390, PMID: 33325020 PMC8026233

[22] Wilkinson D Szekely S Gue B Tam CS Chapman I Heilbronn LK. Assessment of insulin sensitivity during hyperbaric oxygen treatment. Diving Hyperb Med. (2020) 50:238–43. doi: 10.28920/dhm50.3.238-243, PMID: 32957125 PMC7819732

[23] Xu Y Chen J Zhou H Wang J Song J Xie J . Effects and mechanism of stem cells from human exfoliated deciduous teeth combined with hyperbaric oxygen therapy in type 2 diabetic rats. Clinics (Sao Paulo). (2020) 75:e1656. doi: 10.6061/clinics/2020/e1656, PMID: 32520222 PMC7247751

[24] Estrada EJ Valacchi F Nicora E Brieva S Esteve C Echevarria L . Combined treatment of intrapancreatic autologous bone marrow stem cells and hyperbaric oxygen in type 2 diabetes mellitus. Cell Transplant. (2008) 17:1295–304. doi: 10.3727/096368908787648119, PMID: 19364067

[25] Juang JH Hsu BR Kuo CH Ueng SW. Beneficial effects of hyperbaric oxygen therapy on islet transplantation. Cell Transplant. (2002) 11:95–101. doi: 10.3727/096020198389825, PMID: 28853948

[26] Chen SJ Yu CT Cheng YL Yu SY Lo HC. Effects of hyperbaric oxygen therapy on circulating interleukin-8, nitric oxide, and insulin-like growth factors in patients with type 2 diabetes mellitus. Clin Biochem. (2007) 40:30–6. doi: 10.1016/j.clinbiochem.2006.07.007, PMID: 16996047

[27] Estrada EJ Decima JL Bortman G Roberti J Romero EB Samaja G . Combination treatment of autologous bone marrow stem cell transplantation and hyperbaric oxygen therapy for type 2 diabetes mellitus: a randomized controlled trial. Cell Transplant. (2019) 28:1632–40. doi: 10.1177/0963689719883813, PMID: 31665912 PMC6923554

[28] Wang L Zhao S Mao H Zhou L Wang ZJ Wang HX. Autologous bone marrow stem cell transplantation for the treatment of type 2 diabetes mellitus. Chin Med J. (2011) 124:3622–8. 22340214

[29] Xu Q Wei YT Fan SB Wang L Zhou XP. Repetitive hyperbaric oxygen treatment increases insulin sensitivity in diabetes patients with acute intracerebral hemorrhage. Neuropsychiatr Dis Treat. (2017) 13:421–6. doi: 10.2147/NDT.S126288, PMID: 28228657 PMC5312693

[30] Wu Z Cai J Chen J Huang L Wu W Luo F . Autologous bone marrow mononuclear cell infusion and hyperbaric oxygen therapy in type 2 diabetes mellitus: an open-label, randomized controlled clinical trial. Cytotherapy. (2014) 16:258–65. doi: 10.1016/j.jcyt.2013.10.004, PMID: 24290656

